# Plasma-Activated Water Dipping of Fresh-Cut *Eruca sativa*: An Extended Investigation of the Glucosinolate-Myrosinase System and Cellular Responses

**DOI:** 10.3390/foods15193565

**Published:** 2026-10-08

**Authors:** Veronica Lolli, Salihu Mohammed, Luca Calani, Silvia Tappi, Pietro Rocculi, Massimiliano Rinaldi, Ileana Ramazzina

**Affiliations:** 1 Department of Food and Drug, University of Parma, Parco Area delle Scienze 27/A, 43124 Parma, Italy; luca.calani@unipr.it (L.C.); massimiliano.rinaldi@unipr.it (M.R.); 2 Department of Medicine and Surgery, University of Parma, Via Gramsci 14, 43126 Parma, Italy; salihu.mohammed@unipr.it (S.M.); ileana.ramazzina@unipr.it (I.R.); 3 Department of Agricultural and Food Sciences, Alma Mater Studiorum—University of Bologna, Piazza Goidanich 60, 47521 Cesena, Italy; silvia.tappi2@unibo.it (S.T.); pietro.rocculi3@unibo.it (P.R.)

**Keywords:** plasma-activated water, rocket salad, glucosinolates, isothiocyanates, myrosinase, Caco-2 cells, phase II enzymes

## Abstract

Plasma-Activated Water (PAW) is an emerging non-thermal technology with potential applications in fresh-cut vegetable preservation. The present study investigated the effect of PAW dipping on the glucosinolate-myrosinase system of rocket salad (*Eruca sativa*) and on selected cellular responses in human Caco-2 cells. Leaves were dipped in PAW for 20 min (PAW-20) or in untreated water (UT). Glucosinolates (GLSs) and their corresponding isothiocyanates (ITCs) were determined by UHPLC-ESI-MS/MS and GC-MS, while myrosinase activity was assessed in crude enzyme extracts. Caco-2 cells were exposed to extracts derived from PAW-20 or UT rocket leaf samples. Instrumental analyses revealed higher levels of the precursor GLSs, glucoerucin and glucoraphanin, in the PAW-20 sample compared with the UT one, whereas the corresponding ITCs, erucin and sulforaphane, were present at lower levels despite no detectable differences in myrosinase activity. In Caco-2 cells, PAW-20-derived leaf extracts induced the upregulation of the mRNA levels of specific UDP-glucuronosyltransferases (UGTs), while no significant modulation of glutathione S-transferases (GSTs) or the nuclear factor erythroid 2-related factor 2 (Nrf2) mRNA levels was observed. Moreover, only minor changes were detected in inflammation-related target genes. Overall, under the experimental conditions tested, PAW treatment was associated with changes in the glucosinolate-isothiocyanate balance in rocket leaf extracts and with selective modulation of phase II detoxification gene expression in Caco-2 cells.

## 1. Introduction

The consumption of fresh fruits and vegetables is essential for human health, largely due to their rich content of bioactive compounds, which exhibit a wide range of biological activities and may help prevent or mitigate the onset and progression of several diseases [1]. Among vegetables, *Eruca sativa* (rocket salad), a member of the Brassicaceae family, is a valuable source of polyphenols and glucosinolates (GLSs), a class of sulfur-containing secondary metabolites that serve as precursors of biologically active molecules [2]. Indeed, following plant tissue disruption, as occurs during food processing or mastication, the endogenous enzyme myrosinase comes into contact with GLSs, catalyzing their hydrolysis and resulting in the formation of different products, including isothiocyanates (ITCs) [3,4,5]. These compounds have attracted considerable scientific interest due to their potential health-promoting properties and have been investigated for their protective effects against a broad spectrum of diseases, including cancer [6], neuropsychiatric and neurodegenerative disorders [7,8], cardiovascular [8] and inflammatory diseases [9]. Accordingly, preserving these valuable compounds during food processing has encouraged the development of alternative processing technologies aimed at maintaining the nutritional and functional quality of fresh-cut vegetables. In this context, Plasma-Activated Water (PAW) has emerged as a promising chemical-additive-free and non-thermal technology for fresh-cut vegetable processing. PAW generation involves plasma-water interactions, leading to the formation of reactive oxygen and nitrogen species (RONS), changes in pH, and electrical conductivity [10]. The resulting chemical environment has been related to its well-established efficacy in sanitization and microbial decontamination [11]. However, PAW may also influence enzymatic activity [12,13] and modulate plant metabolic pathways, affecting the concentration and profile of bioactive compounds [14]. These effects depend on processing parameters, plant matrix, and storage conditions [12,15].

We previously demonstrated that dipping fresh-cut rocket leaves in PAW for 20 min led to changes in the GLS profile, with higher relative abundances of glucoerucin and glucoraphanin compared with water-dipped leaves. Moreover, we showed that the phytochemical-rich extract obtained from PAW-treated rocket leaves did not induce cytotoxicity in Caco-2 cells and resulted in lower RONS generation compared with the extract from water-dipped leaves [16].

Based on these findings, here we provide an in-depth investigation of the effects of PAW on fresh-cut rocket leaves, both in terms of the phytochemical composition and the biological activity. Specifically, we first investigated the glucosinolate-isothiocyanate balance in rocket leaf extracts by quantitatively determining selected GLSs and their corresponding bioactive ITCs. Myrosinase activity was also assessed to determine whether PAW treatment affected this key step in GLS hydrolysis.

Moreover, the potential implications of consuming PAW-treated foods for human health remain insufficiently investigated. In particular, PAW-induced modifications to the phytochemical profile of food matrices may affect the biological activity typically associated with their bioactive compounds, including their ability to modulate cellular signalling pathways and regulate gene expression [14,15,16]. Therefore, further analyses were performed to investigate selected Caco-2 cell responses after exposure to extracts obtained from PAW-treated rocket leaves. To this end, we focused on the expression of selected genes involved in phase II detoxification and inflammatory responses, which are of particular interest in relation to the effects of the glucosinolate-isothiocyanate system, as documented in a growing body of literature [17,18].

Addressing these knowledge gaps may help support the safe application of PAW technology in food processing and contribute to the development of an appropriate regulatory framework for its future use.

## 2. Materials and Methods

### 2.1. Chemicals

Analytical-grade chemicals, sinigrin (purity ≥ 99%), glucoerucin (purity ≥ 90%), glucoraphenin (purity ≥ 95%), and thioglucosidase from *Sinapis alba* (white mustard) seed (≥100 units/g solid) were purchased from Sigma-Aldrich (St. Louis, MO, USA). Methanol (HPLC grade) was purchased from Romil (Cambridge, UK). Sulforaphane (purity ≥ 90%) and erucin (purity ≥ 98%) standards were obtained from Santa Cruz Biotechnology, Inc. (Dallas, TX, USA).

### 2.2. Raw Material, Handling, and Storage

The leaves of *Eruca sativa* Mill. (Brassicaceae) were purchased from the fresh market (Cesena, Italy), kept under refrigeration (2 ± 1 °C) for approximately 24 h before the experiments. Homogeneous defect-free leaves were selected for each treatment.

### 2.3. Sample Preparation

#### 2.3.1. Plasma-Activated Water (PAW) Generation and Rocket Leaf Treatments

PAW was generated under the operating conditions previously described, yielding specific electrical, physicochemical, and RONS characteristics. Briefly, the Alma Pulse generator (AlmaPlasma s.r.l., Bologna, Italy) operated at a peak voltage of 9 kV, with a fixed frequency of 5 kHz, at 5 mm from the liquid surface of 450 mL of distilled water under continuous stirring. A 4 min activation time was selected as the time required to maximise RONS production, maintaining temperature below 25 °C. The concentration of reactive species was measured during the discharge and in the following 20 min. The Amplex^®^ Red Hydrogen Peroxide Assay Kit (Thermo Fisher Scientific, Waltham, MA, USA) and the Nitrate/Nitrite colorimetric assay (ROCHE, Basel, Switzerland) were used for the measurement of H_2_O_2_ and NO_2_^−^, respectively. The analysis of their concentrations was performed photometrically according to the manufacturer’s protocols using a microplate reader (Rayto Life and Analytical Sciences Co., Ltd., Shenzhen, China). The O_3_ concentration produced in the liquid phase was evaluated according to the manufacturer’s protocols using Chematest 20 (SWAN Analytical Instruments AG, Hinwil, Switzerland). Specifically, H_2_O_2_, NO_2_^−^, and O_3_ concentrations just after generation were 4.5 ± 0.1 mg/L, 30.4 ± 0.9 mg/L, and 0.3 ± 0.1 mg/L, respectively, and decreased progressively due to post-discharge processes as described in [19]. Immediately after the 4 min plasma discharge, 20 g of rocket leaves were dipped in 400 mL of PAW at room temperature for 20 min (product:liquid = 1:20, w:v) under constant agitation. The 20-min dipping time was selected based on the higher total phenolic content and the microbial load reduction previously observed under these treatment conditions [16,19]. After PAW-dipping (PAW-20), rocket leaves were dried on absorbent paper and immediately freeze-dried (24 h, shelf-temperature: −40 °C, pressure: 0.019 mbar). The sample dipped in untreated water (UT) under the same conditions was considered as control. Each treatment was repeated twice to obtain two independent replicates. The freeze-dried samples were stored at −20 °C until analysis.

#### 2.3.2. Preparation of Rocket Leaf Extracts

Methanolic extracts were prepared from each independent replicate, as previously described [16]. Briefly, three g of PAW-20 or UT freeze-dried rocket leaf powder were added to 20 mL of 60% methanol, and vortexed for 2 min. After centrifugation (10,000× *g* for 10 min at 10 °C), the supernatant was collected.

Two independent extractions were performed for each sample, and the supernatants were pooled. After solvent removal, the dried residues were reconstituted in cell culture medium containing 0.5% DMSO (pH 7.1), and the resulting stock solutions were kept at −80 °C until instrumental analysis for GLSs and ITCs determination (see Section 2.4) and cell-based experiments (see Section 2.6).

### 2.4. Instrumental Analysis

#### 2.4.1. Standard Solution Preparation

For UHPLC-ESI-MS/MS analysis, pure standard stock solutions of GLSs (glucoerucin and glucoraphenin) and sulforaphane were prepared in pure methanol at a concentration of 1000 mg/L. Working standard solutions were obtained by diluting the individual standard stock solutions with acidified water (0.2% formic acid) to obtain concentrations ranging from 0.01 to 10 mg/L.

For GC-MS analysis, working standard solutions were prepared by diluting the erucin standard stock solution (1000 mg/L) in dichloromethane (CH_2_Cl_2_; anhydrous, ≥99.8%) to final concentrations ranging from 0.1 to 100 mg/L. UT and PAW-20 spiked samples were prepared by adding an aliquot of erucin standard corresponding to 5 µg of compound.

All the standard solutions were kept in the dark and stored at −20 °C.

#### 2.4.2. UHPLC-ESI-MS/MS Analysis

PAW-20 and UT rocket leaf extracts (described in the previous section) were analysed using a DIONEX Ultimate 3000 UHPLC coupled to a TSQ Vantage triple quadrupole (Thermo Fisher Scientific) equipped with a heated-electrospray ionisation (H-ESI II) probe (Thermo Fisher Scientific). Before analysis, samples were diluted 1:50 with acidified water (0.2% formic acid) to obtain a final concentration of 5 mg/mL of dry extract.

Chromatographic separation was achieved by an ACE UltraCore SuperC18 (2.1 mm i.d. × 100 mm, 2.5 µm particle size, Advanced Chromatography Technologies, Aberdeen, Scotland). The mobile phase consisted of 0.2% formic acid in water (eluent A) and 0.2% formic acid in acetonitrile (eluent B) at a flow rate of 0.35 mL/min. A 9-min linear gradient from 1% to 80% B was applied. The autosampler was kept at 10 °C, while the column compartment was set to 40 °C. The mass spectrometer operated with a vaporiser temperature of 300 °C and a capillary temperature of 270 °C. For glucosinolates, sheath and auxiliary gas (both N_2_) flows were set at 50 and 10 arbitrary units, respectively. Instead, for sulforaphane, the sheath and auxiliary gas were set at 40 and 15 arbitrary units, respectively. For MS/MS analysis, argon was used as the collision gas at a pressure of 1.0 mTorr. Full-scan analysis (*m*/*z* 100–1500) was performed in ESI^−^ mode for glucosinolate analysis and in ESI^+^ mode for sulforaphane determination, as previously described [15]. The spray voltage was 3 kV for ESI^−^ and 3.5 kV for ESI^+^.

Product-ion scan experiments were conducted for compound identification, using a collision energy of 30 V for precursor ions at *m*/*z* 434, 436, and 420 in ESI^−^ mode and at *m*/*z* 178.1 in ESI^+^ mode. Product-ion spectra and chromatographic behavior were compared with those of authentic standards when available. MS experiments in Selected Reaction Monitoring (SRM) mode were carried out to enhance analytical selectivity and sensitivity for component quantification. The precursor-to-product ion transitions, collision energies, and retention times used for each compound are reported in Table S1.

The quantification of targeted compounds was performed using external standard calibration curves (described in Section 2.4.1).

Glucoerucin and sulforaphane were quantified using their corresponding authentic standards and expressed as µmol/L. Glucoraphanin was quantified using a glucoraphenin calibration curve and expressed as µmol glucoraphenin equivalents/L.

For each extract, the UHPLC-ESI-MS/MS analysis was performed in duplicate.

All instrumental data were acquired using Xcalibur software 2.2 (Thermo Fisher Scientific).

#### 2.4.3. GC-MS Analysis

For GC-MS analysis, erucin was recovered from the reconstituted rocket leaf extracts by liquid–liquid extraction with 1 mL of anhydrous dichloromethane (≥99.8%), following a slightly modified protocol [20].

The resulting solution was centrifuged at 11,900 rpm at 4 °C for 5 min. One microliter of the organic phase was then analysed by GC-MS in splitless mode (45 s splitless time). The GC-MS analysis was performed using a 7820A gas chromatograph coupled to a 5977B mass selective detector (Agilent Technologies, Santa Clara, CA, USA) equipped with a J&W DB-5ms capillary column (Agilent Technologies). The oven temperature was programmed from 40 °C to 250 °C at 10 °C/min.

Full-scan acquisition (*m*/*z* 40–550) was performed on the authentic erucin standard to obtain its reference mass spectrum. GC-MS analysis of the rocket leaf extracts was carried out in SIM mode (*m*/*z* 146, 161, 61, and 115) to enhance analytical selectivity and sensitivity for erucin identification and quantification. Erucin identification was confirmed by comparing the retention time and monitored ions in the samples with those of the authentic erucin standard and was further supported by the analysis of spiked UT and PAW-20 extracts.

Quantification was performed using an external calibration curve prepared from erucin standard solutions (described in Section 2.4.1). Erucin concentrations are reported as µmol/L.

For each extract, the GC-MS analysis was performed in duplicate.

All instrumental data were acquired using Agilent ChemStation software C.01.07 (Agilent Technologies).

### 2.5. Myrosinase Activity Assay

To measure myrosinase activity, a previous protocol [21] was adapted with minor modifications. Briefly, frozen chopped rocket leaves obtained from PAW-20 or the UT sample (100 mg) were ground into powder in liquid nitrogen and resuspended in cold 1.5 mL of 33 mM PBS (pH 6.8), containing protease and phosphatase inhibitor cocktails (Sigma-Aldrich). The mixture was vortexed for 30 min at 4 °C and then centrifuged at 14,000× *g* for 30 min at 4 °C. The supernatant was collected to measure myrosinase activity.

Myrosinase activity was determined using a glucose oxidase (GO) assay kit (Sigma-Aldrich), which quantifies the glucose released upon glucosinolate hydrolysis. Briefly, 100 µL of supernatant containing myrosinase from PAW-20 or UT samples was diluted with 30 mM PBS (pH 7.2) to a final volume of 1000 µL to generate the enzyme working solutions. Then, 20 µL of the enzyme working solutions were combined with 880 µL of PBS (pH 7.2) and 100 µL of 2 mM sinigrin or glucoraphenin reference standards dissolved in 30 mM PBS (pH 7.2). The use of two substrates enabled assessment of potential substrate-dependent differences in enzyme activity, as myrosinase isoforms may exhibit different affinities for specific glucosinolates [22].

All samples were incubated at 37 °C for 30 min, and the reaction was stopped by heating at 95 °C for 10 min. Subsequently, 50 µL of the reaction solution was mixed with 100 µL of the GO reagent in a 96-well plate. The reactions were incubated at 37 °C for 30 min and stopped by the addition of 100 µL of 6 M sulfuric acid. Prior to sample analysis, assay performance was verified using a commercial myrosinase standard (Thioglucosidase from *Sinapis alba* seed) over a range of enzyme activities (0.02–1.25 U/mL). A linear dose–response was obtained for both sinigrin (R^2^ = 0.9974) and glucoraphenin (R^2^ = 0.9879), indicating good assay reliability under the selected experimental conditions. Also, a glucose standard curve was prepared in the range of 0.01–0.16 mg/mL. After gentle mixing, the absorbance at 540 nm was measured with the EnSpire^TM^ multimode plate reader (PerkinElmer, Waltham, MA, USA). One unit (U) of myrosinase catalytic activity was defined as the amount of enzyme releasing 1 µmol of glucose per minute at 37 °C. The myrosinase activity of each extract was recalculated per 1 g of fresh weight (fw).

For each treatment, two crude enzyme extracts, corresponding to the two independent processing replicates, were analysed. Each extract was assayed on three separate days, with each determination performed in duplicate wells.

### 2.6. Cell Culture and Treatment

Caco-2 cells were purchased from ATCC^®^ (Manassas, VA, USA) and grown in 1:1 mixture of Ham’s F12:DMEM medium, supplemented with 10% fetal bovine serum (Lonza, Milan, Italy), 2 mM L-glutamine, 100 U/mL penicillin and 100 μg/mL streptomycin, at 37 °C under a 5% CO_2_ atmosphere. Trypsin/EDTA (Sigma-Aldrich) treatment was used for cell harvesting. Rocket leaf extracts from PAW-20 and UT samples were diluted in complete cell medium to the final concentration required for each experiment. Control cells were cultured in medium containing 0.1% DMSO.

Caco-2 cells were treated for 5 h with increasing concentrations of sample extracts, and the expression profiles of phase II detoxification enzyme family members, namely glutathione S-transferase (GSTs) and UDP-glucuronosyltransferase (UGTs), as well as the transcription factor Nrf2, iNOS, and COX-2, were evaluated.

The concentrations tested (0.02, 2, and 20 µM) were defined based on the summed concentration of the polyphenols previously quantified in the methanolic rocket leaf extracts [16].

A 5-h exposure was selected to investigate early transcriptional responses based on previous experiments with food-derived phytochemical extracts [23,24,25,26,27].

### 2.7. RNA Extraction, Reverse Transcription, and Quantitative Real-Time PCR (RT-qPCR)

Caco-2 cells were seeded in a 6-well plate at a density of 6 × 10^5^ and allowed to attach overnight. After treatment with increasing concentrations of diluted extracts (0.02, 2, 20 μM) from PAW-20 or the UT samples described in the section above, total RNA was isolated with the TRIzol™ Reagent (Invitrogen, Carlsbad, CA, USA) and purified with the RNA Clean & Concentrator^TM^-5 Kit (Zymo Research, Irvine, CA, USA) following the manufacturer’s protocol. The RNA obtained was quantified using a spectrophotometer (Eppendorf, Hamburg, Germany) and checked for quality and integrity by electrophoresis. For the reverse transcription reaction, RevertAid Reverse Transcriptase (Thermo Fisher Scientific) was used. For each reaction, 250 ng of total RNA was combined with 1 μL of random primers (0.2 μg μL^−1^, Thermo Fisher Scientific) in a final volume of 12.5 μL, and heated at 65 °C for 5 min. Thereafter, following a brief chill on ice, 4 μL of Reaction Buffer 5X, 2 μL of Deoxynucleotide triphosphate mix (10 mM, Thermo Fisher Scientific), and 1 μL of RevertAid Reverse Transcriptase (200 U μL^−1^) were added, yielding a final volume of 20 μL. Then, the reaction was incubated for 10 min at 25 °C, 60 min at 45 °C, and 10 min at 70 °C. The first strand synthesis reaction was diluted 1:2, and the amplification reactions were performed using 2 μL of cDNA, 10 μL of SsoAdvanced^TM^ Universal SYBR^®^ Green Supermix (Bio-Rad Laboratories, Inc., Hercules, CA, USA), and 0.2 μM of forward and reverse primers (Table S2), in a final reaction volume of 20 μL. Amplifications were performed using the QuantStudio 3 Real-Time PCR Systems (Thermo Fisher Scientific). Relative quantification was calculated using the 2^−ΔΔCT^ method [28], with glyceraldehyde-3-phosphate dehydrogenase (GAPDH) as the housekeeping gene for data normalization. The results are expressed as mean ± standard error of three independent experiments, each performed in duplicate.

### 2.8. Statistical Analysis

For both chemical analyses and myrosinase activity, a two-tailed Student’s *t*-test was performed to compare UT and PAW-20 samples based on the values obtained for each independent processing replicate.

For cell-culture experiments, differences among control, UT, and PAW-20 treatments were evaluated by one-way ANOVA, while direct comparisons between UT and PAW-20 treatments were performed using a two-tailed Student’s *t*-test. Statistical significance level was set at *p* < 0.05. Statistical analyses were performed using SigmaPlot software (version 12.0; Systat Software Inc., San Jose, CA, USA). Given the limited number of independent processing replicates, the statistical comparisons are exploratory and presented to support the interpretation of observed trends.

## 3. Results and Discussion

### 3.1. Effect of PAW on Glucosinolates, Isothiocyanates and Myrosinase Activity

Our previous research showed that the polyphenolic content of fresh-cut *E. sativa* was not affected by 20 min of dipping in PAW (PAW-20) compared with the untreated (UT) sample, whereas a higher relative abundance of glucoerucin and glucoraphanin was observed in the former compared with the latter [16]. Building on these findings, we first investigated the balance between GLS precursors and their corresponding ITC hydrolysis products in the same extracts. To this aim, targeted UHPLC-ESI-MS/MS analysis was performed and revealed higher levels of GLSs, specifically glucoraphanin (870 ± 15 vs. 491.5 ± 40.8 µmol glucoraphenin equivalents/L) and glucoerucin (710 ± 10 vs. 340 ± 20 µmol/L), following PAW dipping (Table 1). These results confirm the trends previously observed based on relative abundance analysis [16].

Then, the corresponding ITCs, namely sulforaphane and erucin, were quantified by UHPLC-ESI-MS/MS and GC-MS, respectively. The results obtained showed a trend opposite to that of their precursor GLSs. Indeed, the concentrations of both ITCs were lower in the PAW-treated extracts than in the UT ones, with reductions exceeding 50%. In particular, sulforaphane and erucin levels decreased following PAW dipping from 365 ± 7 to 134 ± 2 µmol/L and from 10 ± 1 to 4.8 ± 0.5 µmol/L, respectively (Table 1).

The highest GLS levels detected in PAW-20 extract may be attributed to the complex network of reactive species generated during treatment, which can trigger cellular structure disruption and promote the release of bioactive compounds. This effect has been reported for several plasma-based technologies, including PAW [14,29].

Because plasma treatment can also induce the inactivation of different enzymes such as peroxidase, polyphenol oxidase, and catalase [13,14], we hypothesized that, under our experimental conditions, PAW may also result in myrosinase inactivation. Indeed, in healthy plant tissues, myrosinase and GLSs are compartmentalized. However, upon tissue damage, the enzyme can interact with its GLS substrates, allowing the generation of unstable aglycones that can subsequently form ITCs [4,5].

**Figure 1:**
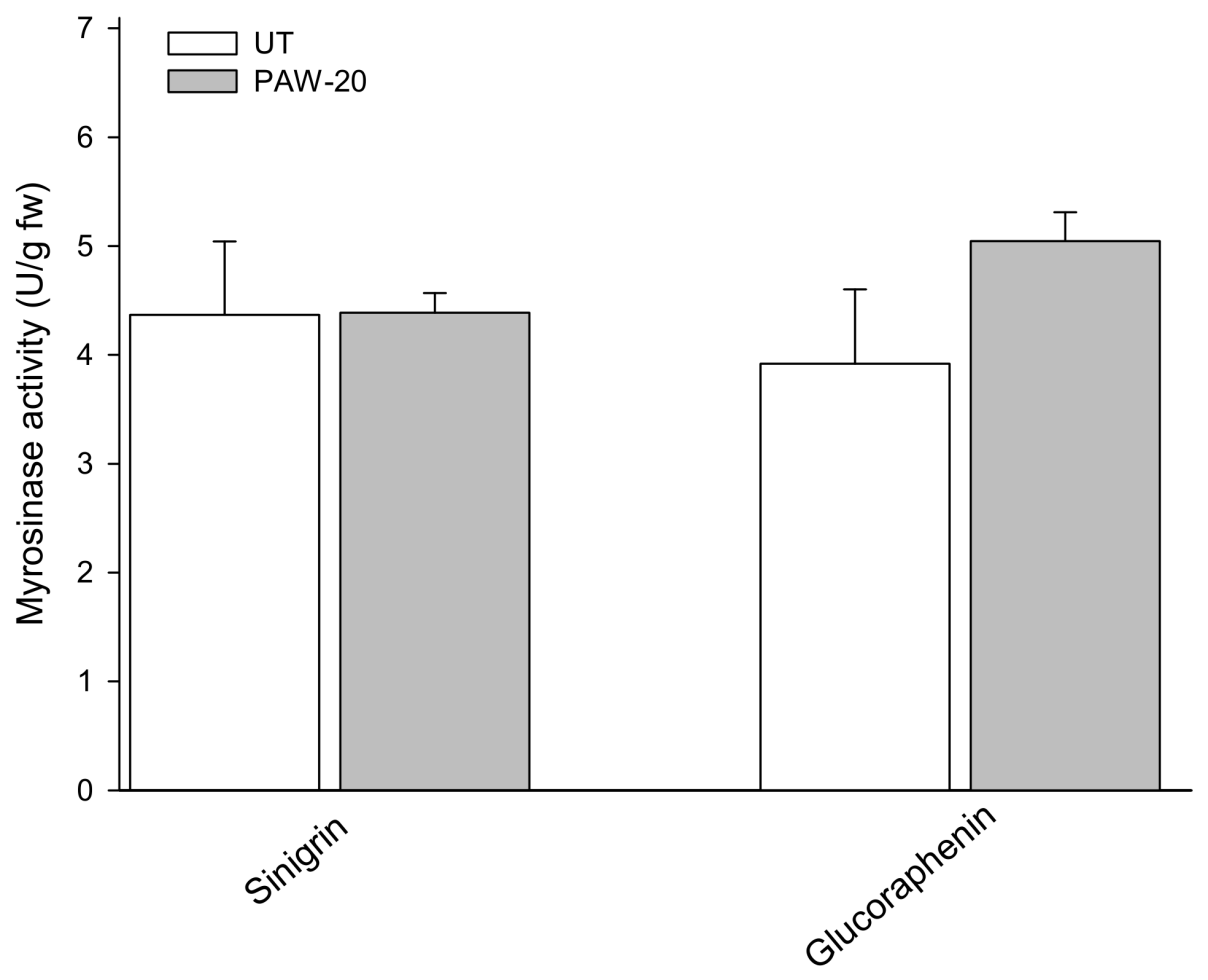
Myrosinase activity (U/g fw) measured in *E. sativa* leaves dipped in untreated water (UT, white bar) or in plasma-activated water for 20 min (PAW-20, grey bar), using either sinigrin or glucoraphenin as reference substrates. Data are presented as means ± standard error from two independent replicates assayed on three separate days, with each determination carried out in duplicate wells. No statistically significant differences were observed between UT and PAW-20 samples for either substrate (*p* ≥ 0.05, two-tailed Student’s *t*-test).

Myrosinase activity was measured in crude rocket leaf extracts obtained from PAW-20 and UT samples, using sinigrin and glucoraphenin, as reference substrates (Section 2.5). Although the myrosinase activity values obtained were in agreement with previously reported data [30], no significant reduction was observed when comparing the PAW-20 and UT samples for either substrate under our experimental conditions (Figure 1).

These results indicate that the high concentrations of glucoraphanin and glucoerucin observed in the PAW-20 extract cannot be attributed to a reduction in myrosinase activity per se, as we initially hypothesized. Nevertheless, it should be considered that the enzymatic assay used measures the enzyme activity after extraction under standardized buffered conditions, which do not reproduce the physicochemical environment within plant tissue during PAW dipping. Since the pro-oxidant environment may alter the chemical structure of sensitive molecules, promoting their degradation and oxidation [29], it is possible that such modifications could generate molecules capable of interfering with enzyme-substrate complex formation, thereby contributing to the observed increase in GLS content. Noteworthy, previous studies have reported that high concentrations of GLS substrates can inhibit myrosinase activity in Brassicaceae species [31,32].

The physicochemical environment generated by PAW could also be considered a factor affecting the ITC levels. Considering the lower content of sulforaphane and erucin detected in PAW-20 extract in comparison to UT one, it is important to underline that myrosinase activity alone does not determine ITC formation. Indeed, after myrosinase-mediated hydrolysis, the unstable aglycone may rearrange to form ITCs or be redirected toward alternative products due to the activity of different specifier proteins, including nitrile-specifier proteins, thiocyanate-forming proteins, and epithiospecifier proteins (ESPs) [4,5]. Although studies elucidating the fate of aglycones into different products are still needed, it is known that pH affects both spontaneous rearrangement of the glucosinolate-derived aglycone and the activity of some ESPs. Indeed, acidic conditions can shift the unstable aglycone away from ITC formation, whereas the activity of individual ESPs also depends mainly on pH and other reaction conditions, such as temperature [33].

Since PAW generated under the conditions used in this study is characterized by pH changes and the presence of RONS species [19], the chemical environment induced by PAW dipping could affect glucosinolate-derived aglycones even without a measurable change in myrosinase activity.

Another hypothesis for the lower ITC levels is related to their inherent chemical instability after formation, which is influenced by environmental factors such as pH, temperature, oxygen availability, and solvent composition [34,35]. Recent evidence also showed that erucin can be oxidized to sulforaphane in mildly acidic vegetable matrices, a conversion further promoted by oxygen exposure, thereby altering the relative balance among ITCs [34]. Moreover, ITCs can react with the nucleophilic groups of biomolecules triggering different signaling pathways [36].

The glucosinolate-isothiocyanate concentration pattern observed in PAW-20 extracts suggests that the effects of PAW dipping extend beyond the direct myrosinase inhibition initially hypothesized. Further in-depth investigations should consider both ITC stability under PAW conditions and the occurrence of GLS hydrolysis products, which could provide evidence for a potential shift in aglycone fate following treatment.

Overall, PAW technology appears to be a promising strategy for increasing GLS content, since they serve as precursors of bioactive compounds such as sulforaphane and erucin. Indeed, despite lower ITC levels detected immediately after treatment, the increased GLS pool may represent a relevant substrate reservoir for subsequent ITC formation during chewing and/or microbial metabolism [17,18], with potential implications for the sensory properties as well as the health-promoting potential of rocket leaves. However, as only the 20-min PAW washing time was evaluated based on previously obtained data [16,19], further investigations are needed to assess the effects of different exposure times on the glucosinolate-isothiocyanate ratio and the stability of these compounds throughout the shelf life of fresh-cut rocket salad.

### 3.2. Effect of PAW Extract on Caco-2 Cells

#### 3.2.1. Expression Profile of UGTs and GSTs

Given that consumption of food rich in polyphenols and ITCs has been shown to exert beneficial effects on human health under both physiological and pathological conditions, we sought to further explore this topic. Specifically, we aimed to investigate whether the pro-oxidant environment induced by PAW generation triggers the formation of compounds in plant tissue that can interfere with the expression profiles of phase II enzymes. Indeed, it is well known that one of the mechanisms ascribable to the beneficial effects of phytochemicals such as sulforaphane is the activation of detoxification pathways [37,38,39].

**Figure 2:**
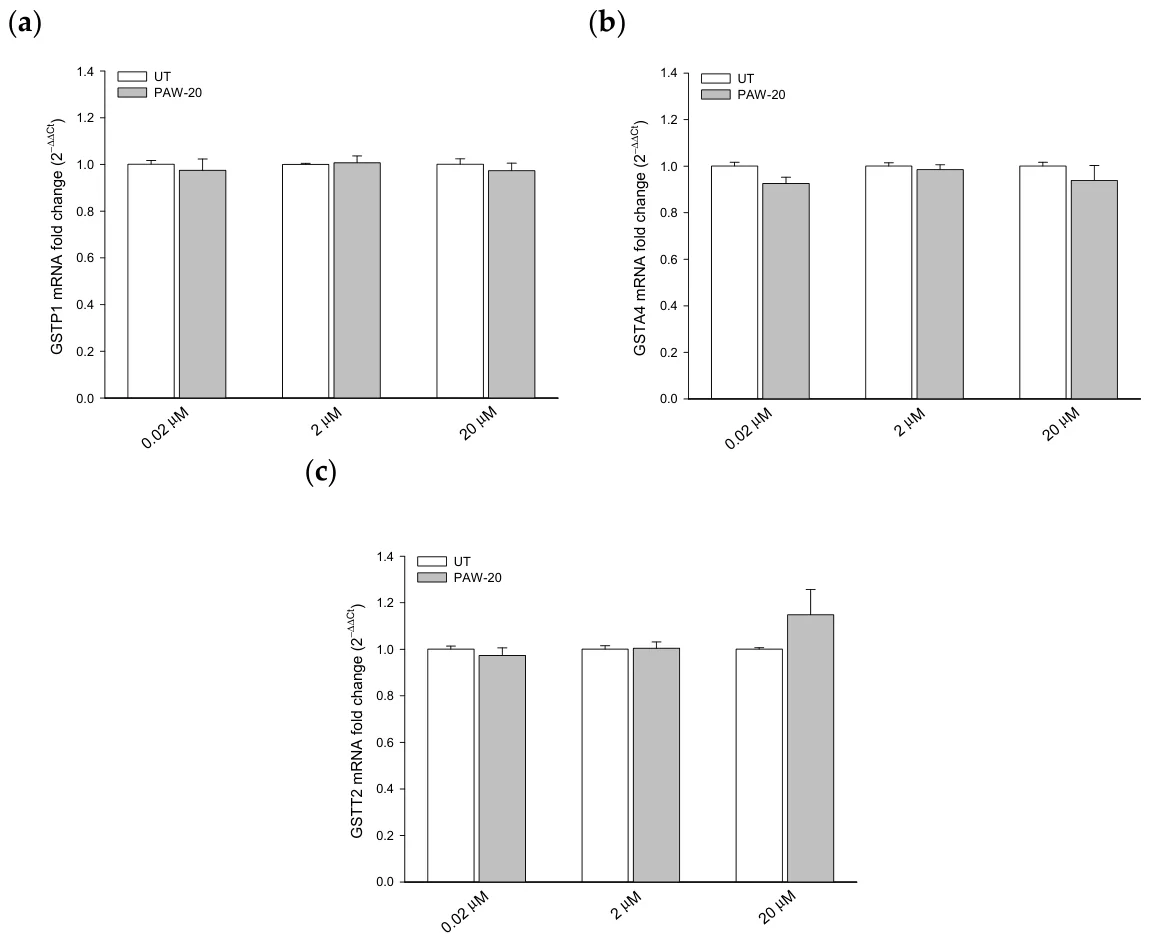
Relative mRNA expression of GST phase II enzymes, (**a**) GSTP1, (**b**) GSTA4, (**c**) GSTT2, evaluated by RT-qPCR. Caco-2 cells were treated for 5 h with increasing concentrations of extracts derived from *E. sativa* leaves dipped in untreated water (UT, white bar) or in plasma-activated water for 20 min (PAW-20, grey bar). Relative quantification was calculated using the 2^−ΔΔCt^ method, with the UT sample as the reference condition (reference mRNA fold change equal to 1). Data are presented as means ± standard error from three independent experiments, each carried out in duplicate. No statistically significant differences were observed between UT and PAW-20 samples for any of the analysed GST genes at any of the tested concentrations (*p* ≥ 0.05, two-tailed Student’s *t*-test).

As reported in the previous section, dipping fresh-cut *E. sativa* leaves in PAW resulted in a significant reduction in sulforaphane and erucin levels compared with dipping in untreated water. Based on these results, a weaker phase II response to the PAW-derived extract was expected. Therefore, we determined the mRNA expression profiles of phase II enzyme family members [40,41], GSTs and UGTs, using RT-qPCR. To this aim, Caco-2 cells were treated for 5 h with increasing concentrations of the PAW-20 or UT extract, ranging from 0.02 to 20 µM of polyphenol-based concentrations, as defined in Section 2.6. As a result, the mRNA levels of GST enzymes, namely GSTP1, GSTA4, and GSTT2, were not significantly changed after exposure to PAW-20 extract compared to the UT one (Figure 2).

Moreover, while treatment with both extracts did not modify the mRNA expression profile of the GSTP1 and GSTA4 target genes compared to control cells, the mRNA level of GSTT2 was slightly but significantly decreased upon treatment with the UT extract at a concentration of 20 µM, compared to both the control cells and the other concentrations tested (Figure S1).

**Figure 3:**
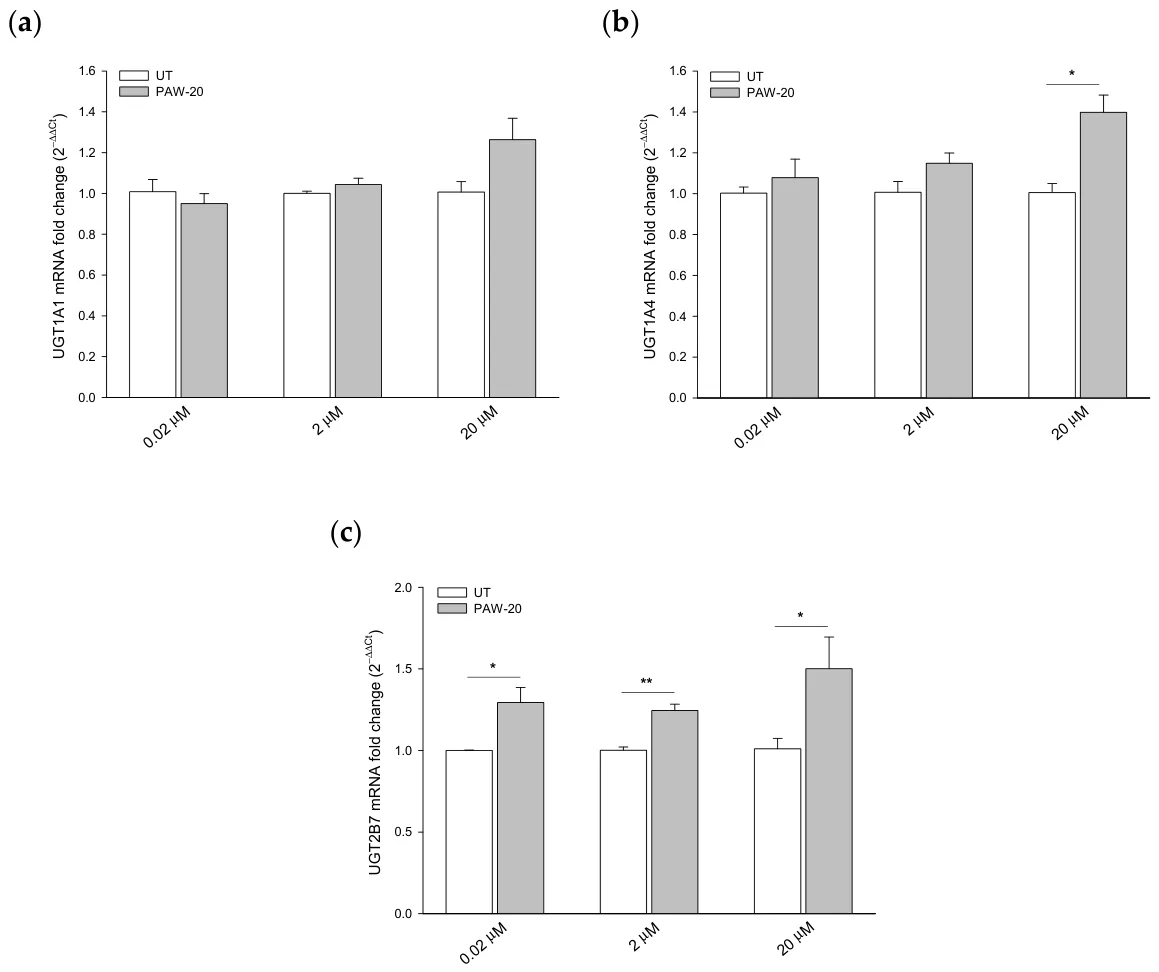
Relative mRNA expression of UGT phase II enzymes, (**a**) UGT1A1, (**b**) UGT1A4, (**c**) UGT2B7, evaluated by RT-qPCR. Caco-2 cells were treated for 5 h with increasing concentrations of extracts derived from *E. sativa* leaves dipped in untreated water (UT, white bar) or in plasma-activated water for 20 min (PAW-20, grey bar). Relative quantification was calculated using the 2^−ΔΔCt^ method, with the UT sample as the reference condition (reference mRNA fold change equal to 1). Data are presented as means ± standard error from three independent experiments, each carried out in duplicate. Statistical significance between UT and PAW-20 treatments was calculated using a two-tailed Student’s *t*-test (* *p* < 0.05; ** *p* < 0.001).

In contrast, a significant increase in mRNA expression of UGT1A4 and UGT2B7 was observed in cells treated with PAW-derived phytochemical-rich extracts compared with the UT one (Figure 3).

Treatment of Caco-2 cells with both extracts resulted in a significant concentration-dependent increase (*p* < 0.05) in UGT1A1 and UGT1A4 mRNA levels compared with control cells. However, the mRNA level of the UGT2B7 gene was significantly reduced by the treatment with the UT extract at the concentrations tested (Figure S2).

In particular, no broad or consistent effects on GST gene expression were observed, whereas selective modulation of specific UGT genes was detected, contrary to what would have been expected given the reduced sulforaphane content. This result suggests that phase II enzyme adaptive response does not depend solely on sulforaphane concentration but may instead reflect the combined effect of multiple phytochemicals and/or PAW-induced chemical modifications within the extract. Notably, similar differential responses among phase II enzymes have been previously reported in Caco-2 cells treated with pure prenylflavonoids [42], supporting the notion that phase II enzyme families may respond differently to phytochemical exposure.

Identifying the specific phytochemicals responsible for UGT induction and elucidating the molecular mechanisms underlying this regulation will require targeted mechanistic investigations, which represent an interesting topic for future studies.

#### 3.2.2. Expression Profile of Nrf2

**Figure 4:**
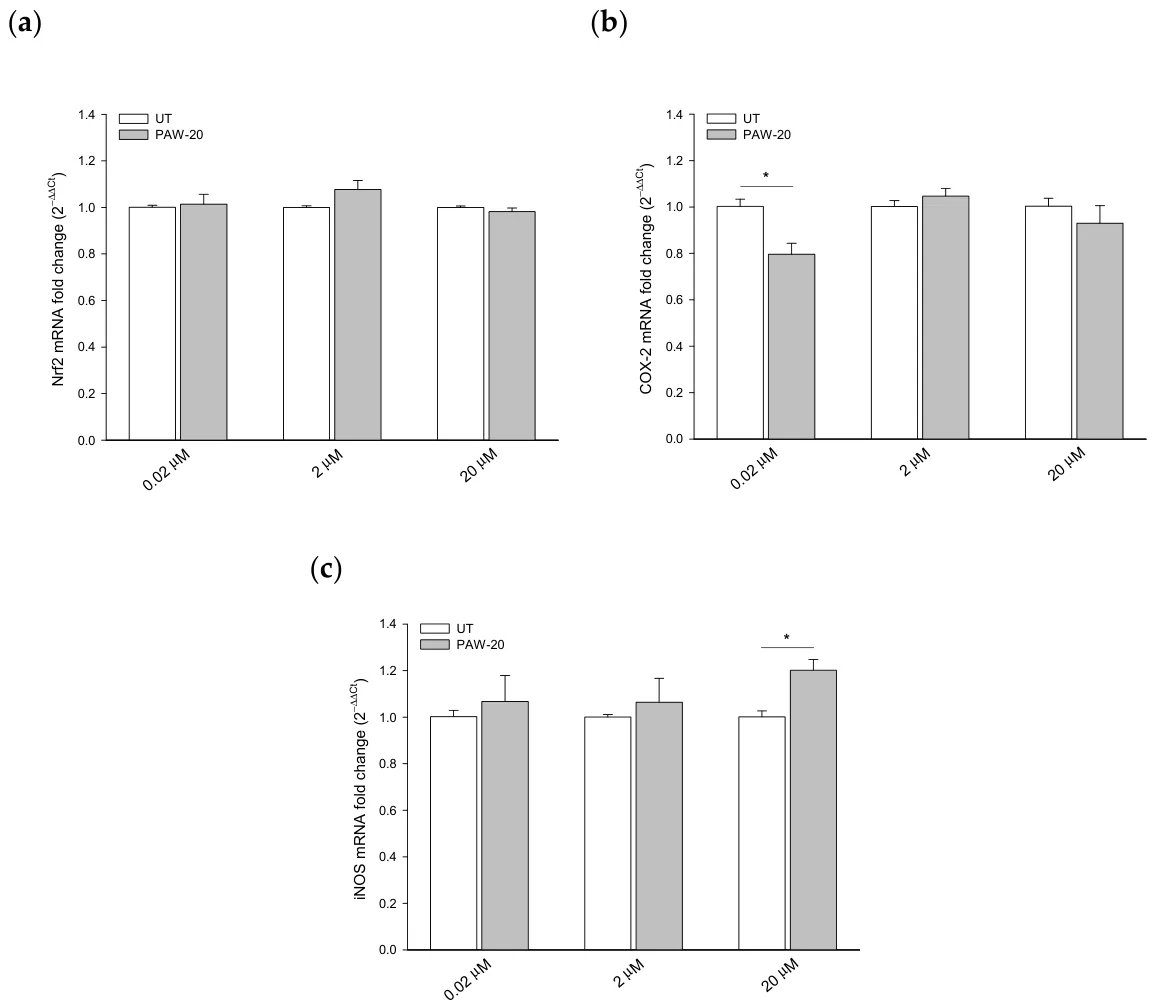
Relative mRNA expression of (**a**) Nrf2, (**b**) COX-2, and (**c**) iNOS evaluated by RT-qPCR. Caco-2 cells were treated for 5 h with increasing concentrations of extracts derived from *E. sativa* leaves dipped in untreated water (UT, white bar) or in plasma-activated water for 20 min (PAW-20, grey bar). Relative quantification was calculated using the 2^−ΔΔCt^ method, with the UT sample as the reference condition (reference mRNA fold change equal to 1). Data are presented as means ± standard error from three independent experiments, each carried out in duplicate. Statistical significance between UT and PAW-20 treatments was calculated using a two-tailed Student’s *t*-test (* *p* < 0.05).

Nrf2 is a key regulator of the cellular defense system, controlling the expression of genes involved in antioxidant response, cofactor regeneration, and xenobiotic metabolism, including phase II enzymes [43,44]. Accordingly, we also analysed whether PAW-20 extract could alter its mRNA expression profile. Our data showed no significant differences in the effects of PAW-20 and UT extracts (Figure 4). This finding is consistent with previous evidence indicating that phytochemicals such as polyphenols and ITCs primarily regulate Nrf2 at the post-translational level rather than through transcriptional upregulation [45,46]. However, protein expression was not investigated in the present study. The comparison with control cells did not reveal any statistically significant modulation of Nrf2 mRNA levels following treatment with either extract (Figure S3).

#### 3.2.3. Expression Profile of iNOS and COX-2

Considering that Nrf2 is also involved in the regulation of inflammatory pathways [43,46] and that intestinal epithelial cells represent the first line of defense in the gastrointestinal tract [47], we also explored the mRNA levels of the inducible isoform of Nitric Oxide Synthase (iNOS) and Cyclooxygenase-2 (COX-2). The former is responsible for the production of large amounts of nitric oxide [48], whereas the latter is involved in the metabolism of prostanoids [49]. Under the tested experimental conditions, the treatment of Caco-2 cells with PAW-20-derived extract resulted in a statistically significant reduction in the mRNA level of COX-2 at the lowest concentration tested (0.02 μM) and in a statistically significant increase in the iNOS mRNA level at the highest concentration (20 μM) when compared to the UT sample (Figure 4).

However, comparison with control cells did not reveal any consistent modulation of COX-2 and iNOS transcription (Figure S3).

The absence of a clear anti-inflammatory effect, despite this is well documented in the literature for polyphenolic compounds [50] and ITCs, including sulforaphane [51], may reflect the absence of inflammatory stimuli, such as lipopolysaccharide or cytokine treatment. Indeed, this study did not include a simulated inflammatory environment and was limited to a single-cell model. Because the intestinal barrier consists of multiple cell types, including immune cells from both the innate and adaptive immune systems [52,53,54], further studies are needed to explore in depth the effects of PAW-derived extracts in experimental settings mimicking the human gut (e.g., co-culture model of Caco-2 and macrophages).

## 4. Conclusions

The effectiveness and safety of PAW technology in the food industry remain active areas of research, particularly regarding its effects on bioactive compounds and the potential formation of harmful chemical species.

Under the experimental conditions tested in this study, PAW dipping resulted in changes in the glucosinolate-isothiocyanate balance of *E. sativa* leaf extract. Moreover, when tested in Caco-2 cells, the same extract selectively modulated the mRNA expression of genes encoding phase II detoxification enzymes, whereas no marked changes in inflammation-related gene expression were observed.

Further studies are needed to elucidate the mechanisms underlying PAW-induced changes in the phytochemical profile and to investigate the effects of acute and chronic exposure of intestinal epithelial cells to bioactive compounds derived from PAW-treated food matrices. This would provide useful information on the biological relevance of PAW-induced phytochemical changes and provide further evidence to support the safety assessment of PAW-treated foods.

## Figures and Tables

**Table 1:** Glucosinolate (GLS) and isothiocyanate (ITC) concentration (µmol/L) ^1^ in methanolic extracts of rocket leaves obtained after dipping in untreated water or in PAW for 20 min.

Compound	UT	PAW-20
**GLSs**		
Glucoraphanin ^2^	491.5 ± 40.8 ^a^	870 ± 15 ^b^
Glucoerucin	340 ± 20 ^a^	710 ± 10 ^b^
**ITCs**		
Sulforaphane	365 ± 7 ^b^	134 ± 2 ^a^
Erucin	10 ± 1 ^b^	4.8 ± 0.5 ^a^

^1^ Data are expressed as mean ± standard deviation of two independent dipping procedures, each analysed in technical duplicate. ^2^ Glucoraphanin was quantified using a glucoraphenin calibration curve and is expressed as µmol glucoraphenin equivalents/L. Different letters within the same row indicate statistically significant differences (two-tailed Student’s *t*-test, *p* < 0.05).

## Data Availability

The original contributions presented in this study are included in the article. Further inquiries can be directed to the corresponding author.

## Supplementary Materials

The following supporting information can be downloaded at https://www.mdpi.com/article/10.3390/foods15193565/s1. Figure S1: Relative mRNA expression of GST phase II enzymes evaluated by RT-qPCR. Caco-2 cells were treated for 5 h with increasing concentrations of extracts derived from *E. sativa* leaves dipped in untreated water or in plasma-activated water for 20 min. Relative quantification was calculated using Caco-2 cells incubated in culture medium containing 0.1% DMSO as a reference sample (reference mRNA fold change equal to 1); Figure S2: Relative mRNA expression of UGT phase II enzymes evaluated by RT-qPCR. Caco-2 cells were treated for 5 h with increasing concentrations of extracts derived from *E. sativa* leaves dipped in untreated water or in plasma-activated water for 20 min. Relative quantification was calculated using Caco-2 cells incubated in culture medium containing 0.1% DMSO as a reference sample (reference mRNA fold change equal to 1); Figure S3: Relative mRNA expression of Nrf2, COX-2, and iNOS evaluated by RT-qPCR. Caco-2 cells were treated for 5 h with increasing concentrations of extracts derived from *E. sativa* leaves dipped in untreated water or in plasma-activated water for 20 min. Relative quantification was calculated using Caco-2 cells incubated in culture medium containing 0.1% DMSO as a reference sample (reference mRNA fold change equal to 1); Table S1: Selected Reaction Monitoring (SRM) parameters and retention times used for the UHPLC-ESI-MS/MS determination of glucosinolates and sulforaphane. The reported retention time represents the typical retention time observed for glucoraphanin in rocket leaf extracts under previously reported analytical conditions [16]; Table S2: Sequences of the primers used in qPCR analysis, as previously reported [40,55,56,57,58].

## Abbreviations

The following abbreviations are used in this manuscript:

GLSs	Glucosinolates
ITCs	Isothiocyanates
PAW	Plasma-Activated Water
UT	Untreated water
RONS	Reactive Oxygen and Nitrogen species
Nrf2	Nuclear Factor Erythroid 2-Related Factor 2
NF-kB	Nuclear Factor kappa B
iNOS	Inducible Nitric Oxide Synthase
COX-2	Cyclooxygenase-2
ESPs	Epithiospecifier Proteins
