# Supplementary material for: Plasma-Activated Water Dipping of Fresh-Cut Eruca sativa: An Extended Investigation of the Glucosinolate-Myrosinase System and Cellular Responses
Source: Foods. 2026 Oct 8;15(19):3565. doi: 10.3390/foods15193565 (PMC13664880; doi:10.3390/foods15193565)
Supplement: Supplementary file 1 [file foods-15-03565-s001.zip › Figure S3.pdf]

Supplementary Figure S3

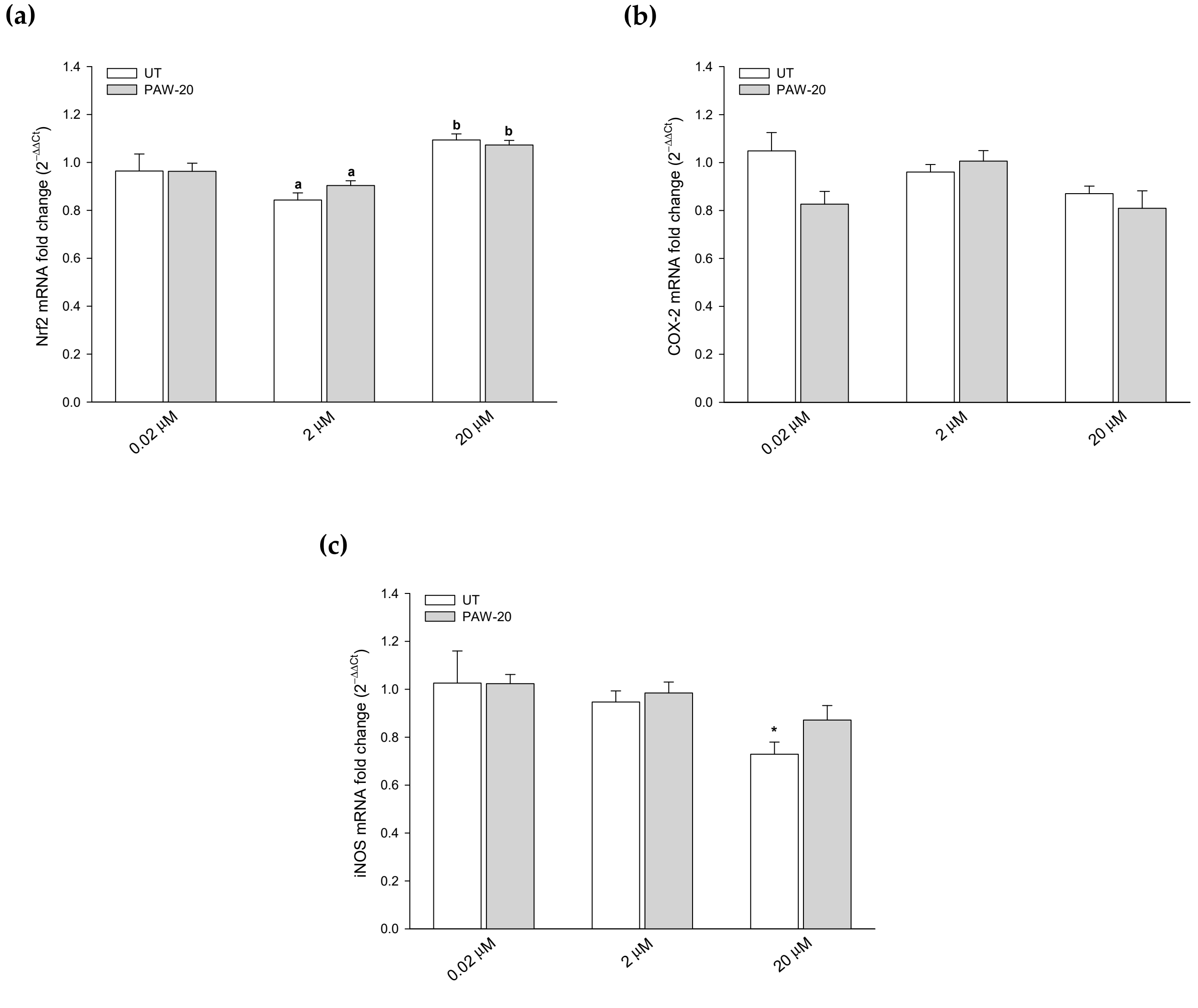

Supplementary Figure S3. Relative mRNA expression of (a) Nrf2, (b) COX-2, and (c) iNOS evaluated by RT-qPCR. Caco-2 cells were treated for 5 h with increasing concentrations of extracts derived from *E. sativa* leaves dipped in untreated water (UT, white bar) or in plasma-activated water for 20 min (PAW-20, grey bar). Relative quantification was calculated using the $2^{-\Delta\Delta Ct}$ method using Caco-2 cells incubated in culture medium containing 0.1% DMSO as a reference sample (reference mRNA fold change equal to 1). Data are presented as means ± standard error from three different experiments, each carried out in duplicate. Statistical significance was calculated by pairwise multiple comparisons for one-way ANOVA, followed by Tukey's HSD post hoc test for each extract. *: significant difference versus control, $p < 0.05$. Different letters, when reported, indicate a significant difference among the concentrations tested within each group, $p < 0.05$.
