# Supplementary material for: Plasma-Activated Water Dipping of Fresh-Cut Eruca sativa: An Extended Investigation of the Glucosinolate-Myrosinase System and Cellular Responses
Source: Foods. 2026 Oct 8;15(19):3565. doi: 10.3390/foods15193565 (PMC13664880; doi:10.3390/foods15193565)
Supplement: Supplementary file 1 [file foods-15-03565-s001.zip › Table S2.pdf]

**Table S2.** Sequences of the primers used in qPCR analysis.

| Gene (Gene ID) | Primer Forward 5′→3′ | Primer Reverse 5′→3′ | Amplicone size (pb) | Annealing temperature (°C) | Reference |
|---|---|---|---|---|---|
| **Nrf2 (4780)** | CATCCAGTCAGAAACCAGTGG | GCAGTCATCAAAGTACAAAGCAT | 85 | 61 | Yang et al., 2014 [55] (doi: 10.1186/1476-4598-13-48) |
| **COX-2 (5743)** | ATTGACCAGAGCAGGCAGAT | CTCCACAGCATCGATGTCAC | 153 | 61 | Wang et al., 2022 [56] (doi: 10.3390/antiox11081627) |
| **iNOS (4843)** | ACAGCACATTCAGATCCCCA | GCCGAGATTTGAGCCTCATG | 155 | 61 | Wang et al., 2022 [56] (doi: 10.3390/antiox11081627) |
| **GSTP1 (2950)** | GGAGACCTCACCCTGTACCA | CTGCTGGTCCTTCCCATAGA | 81 | 60 | Scharmach et al., 2009 [57] (doi: 10.1016/j.tox.2009.09.017) |
| **GSTT2 (2953)** | CTTTCCTGGGTGCTGAGCTA | GGTGTTGGGAGGGTTTTCTT | 88 | 60 | Scharmach et al., 2009 [57] (doi: 10.1016/j.tox.2009.09.017) |
| **GSTA4 (2941)** | TCCGTGAGATGGGTTTTAGC | GGTGGTTACCATCCTGCAAC | 97 | 60 | Scharmach et al., 2009 [57] (doi: 10.1016/j.tox.2009.09.017) |
| **UGT1A1 (54658)** | AATAAAAAAGGACTCTGCTATGCT | ACATCAAAGCTGCTTTCTGC | 96 | 60 | Ohno et al., 2009 [40] (doi: 10.1124/dmd.108.023598) |
| **UGT1A4 (54657)** | GAACAATGTATCTTTGGCCC | ACCACATCAAAGGAAGTAGCA | 96 | 60 | Ohno et al., 2009 [40] (doi: 10.1124/dmd.108.023598) |
| **UGT2B7 (7364)** | GGAGAATTTCATCATGCAACAGA | CAGAACTTTCTAGTTATGTCACCAAATATTG | 123 | 60 | Ohno et al., 2009 [40] (doi: 10.1124/dmd.108.023598) |
| **GAPDH (2597)** | AACCTGCCAAATATGATGAC | TTGAAGTCAGAGGAGACCAC | 109 | 60 | Bonacini et al., 2015 [58] (doi:10.1016/j.bbagrm.2014.11.003) |
